# Developing a prediction model for poor prognosis in MPA patients using initial admission examination results: a machine learning study from Southwest China

**DOI:** 10.3389/fimmu.2026.1820073

**Published:** 2026-07-01

**Authors:** Naidan Zhang, Jianfei E., Sijing Ren, Hualiang Xiao, Yuzhi Wang, Xiao Bao, Chengliang Yuan

**Affiliations:** 1 Department of Laboratory Medicine, Peoples Hospital of Deyang City, Deyang, China; 2 Department of Rheumatology, Peoples Hospital of Deyang City, Deyang, China

**Keywords:** adverse prognosis, age, immunosuppressants, initial admission examination, machine learning, microscopic polyangiitis

## Abstract

**Background:**

Microscopic polyangiitis (MPA) is one of the main types of ANCA-associated vasculitis (AAV), but current admission examination indicators are limited in predicting poor prognosis for MPA. This study aims to develop a prediction model for adverse prognosis in MPA patients using initial admission examination results.

**Methods:**

We performed machine learning (ML) algorithms on initial admission examination data to predict adverse outcomes in MPA, such as in-hospital death or self-discharge due to critical condition. We analyzed data from 12,497 patients who underwent ANCA tests in Deyang People’s Hospital between November 2017 and October 2025, focusing on 230 hospitalized patients. We used least absolute shrinkage and selection operator (LASSO), logistic regression (LR), and random forest (RF) to select variables, and evaluated the diagnostic efficacy using ML algorithms. SHapley Additive exPlanations (SHAP) was used for model interpretability. A nomogram model was developed to predict adverse outcomes, highlighting variable contributions and including calibration and decision curve analysis (DCA) curves.

**Results:**

In this study of 230 MPA patients, 56 had poor prognosis while 174 had good prognosis. Seven indicators were identified using LASSO, LR and RF. They were appropriate use of immunosuppressants, age, serum albumin, infection, BVAS, C-reactive protein (CRP) and anti- myeloperoxidase. Among five ML models, support vector classification (SVC) had a high area under the curve (AUC) (AUC = 0.848, 95% confidence interval [CI]: 0.684-0.965) in the prediction model, and had the highest AUC (AUC = 0.886, 95% CI: 0.741-0.981). The SHAP analysis highlighted elevated CRP levels as the top predictor of poor prognosis. Standardized immunosuppressive therapy was found to mitigate this risk. Nomogram model confirmed these findings, and calibration and DCA curves showed this model was reliable and useful for clinical decisions.

**Conclusions:**

We developed a prediction model for adverse outcomes in MPA patients, utilizing clinical and laboratory data collected on the day of admission. SVC algorithm exhibited moderate predictive efficacy. Factors such as elevated serum CRP levels, moderate reductions in serum albumin, infection, advanced age, BVAS larger than 15, and anti-MPO were positively associated with adverse prognoses. Standardized immunosuppressive therapy was shown to mitigate this risk, offering a valuable reference for clinical decision-making.

## 1 Introduction

Microscopic polyangiitis (MPA) represents a predominant form of anti-neutrophil cytoplasmic antibodies (ANCA)-associated vasculitis (AAV), particularly within the Asians. Epidemiological research indicates that the incidence and prevalence of MPA among adults are higher than those of granulomatosis with polyangiitis (GPA), comprising over 90% of AAV cases (1). With advancements in ANCA classification criteria, the enhanced application of serological assays for ANCA and heightened clinical awareness, there has been a notable increase in the incidence and prevalence of MPA. Furthermore, the peak age of onset has shifted significantly towards older adults (2). Studies have demonstrated that a later age at diagnosis correlates with a poorer prognosis for AAV (3, 4). Despite considerable advancements in the standardization of diagnostic criteria for AAV, research on prognostic indicators remains limited.

In the evaluation of the AAV prognosis, there are several challenges. Firstly, there are no indicators directly linked to the prognosis of AAV. Although anti-myeloperoxidase (anti-MPO) antibody plays a crucial role in the diagnosis of MPA, it does not have a direct correlation with the poor prognosis of MPA (5, 6). Secondly, elderly AAV patients often coincide with other comorbidities. It is complicating the clinician’s ability to ascertain which condition predominantly influences the patient’s prognosis. Furthermore, in comparison to rheumatoid arthritis (RA) and systemic lupus erythematosus (SLE), the prevalence of AAV remains low. This scarcity results in considerable variability in findings across different research institutions. Although some studies have developed models based on clinical and laboratory data from hospitalized AAV patients, the predictive efficacy remains limited (7, 8). Therefore, there is a pressing need to identify reliable prognostic indicators and enhance predictive models for MPA.

As large databases continue to evolve, machine learning (ML) is gaining more attention in clinical diagnosis and prognosis assessment. One study developed a ML model using clinical data to predict chronic fatigue in GPA patients, enhancing diagnostic capabilities in areas with limited specialist resources (9). Another study created eight ML models using local data for diagnosing non-purpura type abdominal immunoglobulin A vasculitis, aiding clinical decision-making (10). Currently, there are few studies on predicting the prognosis of MPA in southwestern China. This study aims to construct ML models based on initial examination results to predict adverse prognosis and evaluate their effectiveness, providing a quicker and more convenient tool for clinical decision-making.

## 2 Materials and methods

### 2.1 Research process

This study was a single-center, retrospective study in Deyang People’s Hospital. It encompassed data from 12,497 patients who underwent ANCA tests between November 2017 and October 2025. A total of 230 hospitalized patients diagnosed with MPA were included. The cohort comprised 114 males and 116 females, all of whom were Han Chinese residents from the southwestern region of China. The diagnosis of MPA adhered to the 2012 Chapel Hill Consensus Conference (CHCC) classification criteria. ANCA indicators were assessed using positive results from enzyme immunoassay or chemiluminescence methods for anti-MPO and anti-proteinase 3 (anti-PR3). If the patients had additional disease, MPA was considered the primary diagnosis for the hospitalization. Characteristics and initial laboratory data, including blood routine examination, liver and kidney function examinations were extracted from medical records. In this study, we employed temporal validation as a pre-external validation. The dataset was organized chronologically based on the admission dates of patients and subsequently partitioned into a training set and a validation set using a 7:3 ratio. Specifically, 161 patients admitted between November 2017 and April 2022, constituted the training set. While 69 patients admitted from May 2022 to October 2025, formed the validation set. This methodology was designed to optimally simulate the application of the predictive model on prospective patient cohorts (11). Informed consent for hospital treatment was obtained from each patient upon admission. Since only anonymous registration data were analyzed, no additional consent forms related to this analysis were required. The study was approved by the Ethics Committee of Deyang People’s Hospital (2024-04-037-K01). All methods were carried out in accordance with relevant regulations and in accordance with the Helsinki Declaration.

### 2.2 Inclusion criteria and exclusion criteria

The inclusion criteria were specified as follows (1): participants were aged 18 years or older (2); they must be of Han ethnicity and have resided in the southwestern region of China for an extended period (3); all diagnoses must adhere to the 2012 CHCC classification criteria and be confirmed by rhumatologue (4); assessment of ANCA was primarily based on the results obtained from enzyme immunoassay or chemiluminescence methods for anti-MPO and anti-PR3. In cases where ANCA results were negative, histopathological evidence served as an auxiliary diagnostic tool (5); all relevant hematological tests must be collected within four hours of patient admission. Special reports including antinuclear antibodies (ANAs), anti-cyclic citrullinated peptide (anti-CCP) antibody were issued within 72 hours after receiving the sample.

**Figure 1:**
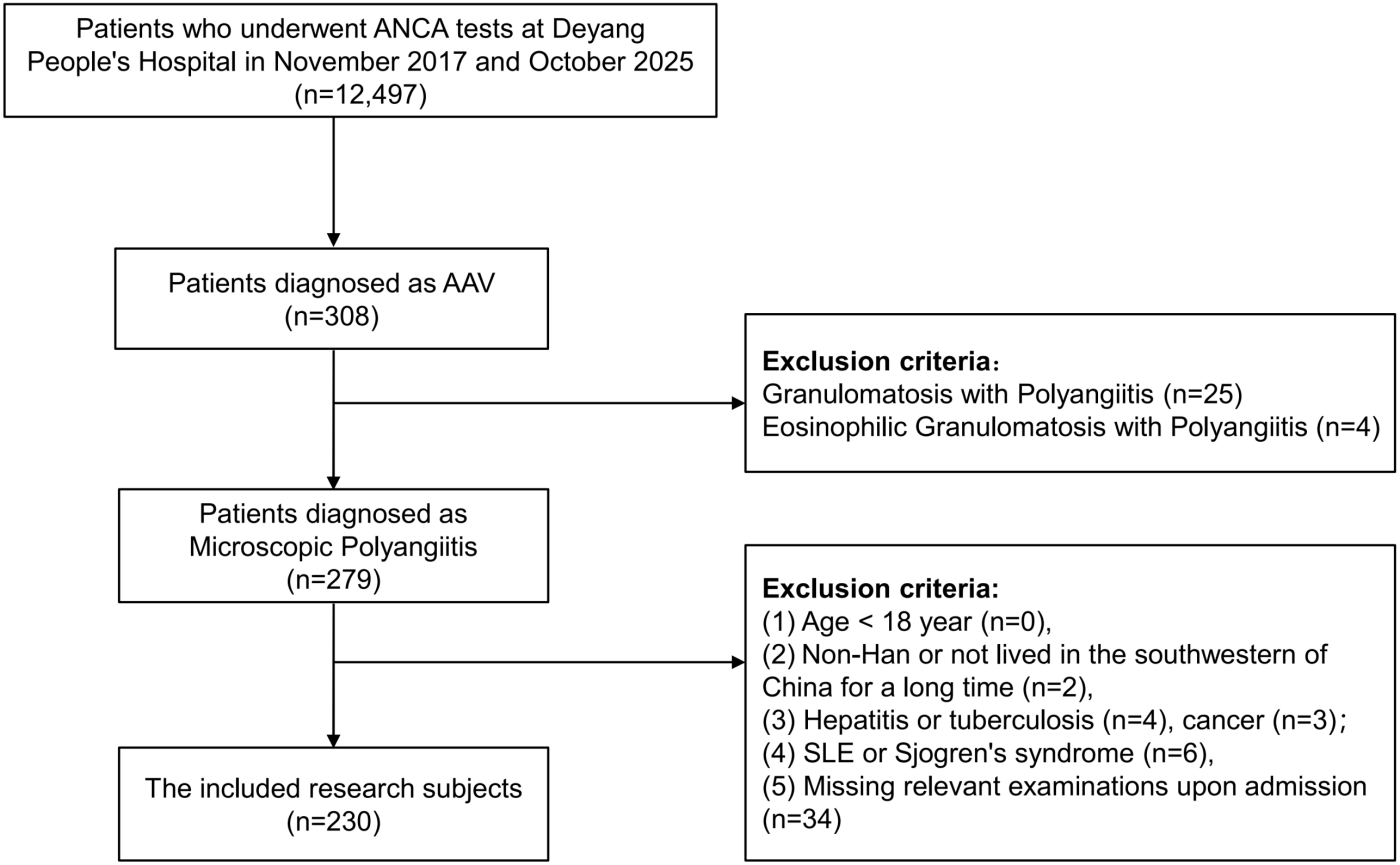
Flowchart of the inclusion criteria for the research. ANCA, anti-neutrophil cytoplasmic antibodies; AAV, ANCA-associated vasculitis; SLE, systemic lupus erythematosus.

The exclusion criteria were delineated as follows: (1) individuals below the age of 18 years; (2) individuals of non-Han ethnicity or those who had not resided in the southwestern region of China for an extended period; (3) individuals diagnosed with infectious diseases (including hepatitis and tuberculosis), drug-related conditions, or neoplastic diseases that would result in secondary vasculitis; (4) patients diagnosed with SLE or Sjögren’s syndrome. (5) AAV individuals who have not undergone the requisite hematological assessments within four hours post-admission. The flowchart of the inclusion criteria for the research subjects is shown in **Figure 1**.

### 2.3 Definitions of comorbidities and adverse outcomes

The complications examined in this study predominantly encompassed conditions lacking a direct causal association with MPA, such as hypertension and type 2 diabetes. Hypertension was characterized by a systolic blood pressure of ≥ 140 mm Hg and/or a diastolic blood pressure of ≥ 90 mm Hg, as measured on the day of admission. Blood pressure assessments were conducted by trained and qualified nursing personnel utilizing a calibrated electronic sphygmomanometer. Additionally, patients with a confirmed prior diagnosis of hypertension who were undergoing antihypertensive treatment were included in the study. Type 2 diabetes was defined as either a new diagnosis made during the admission or as patients who were already on medication for blood sugar management prior to admission.

The adverse outcomes primarily encompass two aspects: (1) during the hospitalization, the patient suffered cardiac and respiratory arrest, cessation of central nervous system activity, and was pronounced deceased following unsuccessful resuscitation efforts by the clinical team; (2) patient’s condition remained critical, Birmingham vasculitis activity score (BVAS) increased and showed no improvement despite treatment interventions, leading the patient’s family to request an early discharge. The criteria for determining a critical condition were based on a Modified Early Warning Score (MEWS) of ≥ 5 points. The evaluation of all adverse outcomes was conducted by specialists in rheumatology and immunology, with information sourced from the patient’s discharge records for this hospitalization.

### 2.4 Collection of clinical and laboratory data

Baseline information including age, gender, diabetes, blood pressures, smoking history, standardized immunosuppressive therapy (S-IST) were collected. Estimated pulse wave velocity (ePWV) was calculated using the initial systolic and diastolic blood pressure (12). The formula for ePWV was as follows: ePWV = 9.587 - 0.402 × age + 4.560 × 10^-3^ × age^2^ - 2.621 × 10^-5^ × age^2^ × MBP + 3.176 × 10^-3^ × age × MBP - 1.832 × 10^-2^ × MBP, where the mean arterial pressure (MBP) was defined as diastolic pressure (DBP) plus 0.4 times the difference between systolic pressure (SBP) and diastolic pressure. According to the World Health Organization’s 1997 definition, smoker was classified as he/she had engaged in smoking continuously or cumulatively for six months or more (13). Ex-smoker was considered if he/she had abstained from smoking for a period exceeding two years at the time of hospital admission. Quantity of smoking was quantified using the smoking index (SI). The formula for SI was as follows: SI = number of cigarettes × years of smoking. SI of 200 or less denoted light smoking. SI between 200 and 400 signified moderate smoking. And SI of 400 or more indicated heavy smoking.

AAV was a progressive autoimmune disease. If not treated in time, it could cause irreversible organ damage and rarely showed spontaneous remission. Consequently, it was imperative to initiate induction therapy immediately following the diagnosis of AAV. The treatments for AAV were bifurcated into two distinct phases: induction remission and maintenance remission (14). In this study, S-IST was the treatment protocol recommended by the European League Against Rheumatism (EULAR) published in 2009 (15). Non-standardized immunosuppressive therapy (NS-IST) included two scenarios. The first scenario involved patients who declined to adhere to the recommended regimen of adequate hormone combined with immunosuppressants during the induction remission. The second scenario involved patients who reduced the dose of glucocorticoids without following the doctor’s instructions during the maintenance remission. To determine if a patient was on standard immunotherapy before admission, we considered two main factors. During induction remission, we checked timely receipt of immunosuppressants like rituximab or cyclophosphamide, ignoring cumulative drug doses. During maintenance remission, we focused on whether patients reduced hormone doses without the doctor’s instructions.

Laboratory data encompassed the initial blood routine, liver and kidney function, procalcitonin (PCT), C-reactive protein (CRP) and d-dimer following patient admission. Blood routine included white blood cell (WBC) count, absolute neutrophil count, absolute lymphocyte count, absolute monocyte count, hemoglobin (HGB) and platelet count. Blood routine was performed using the Sysmex XN-1000 blood analyzer (Sysmex, Japan). These analyses along with the appropriate reagents. Liver and kidney function including total protein, albumin, serum creatinine, urea, uric acid and cystatin C, measured by the ADVIA 2400 automated biochemical analyzer (Siemens, Germany). The estimated glomerular filtration rate (eGFR) for this region was determined utilizing the Chronic Kidney Disease Epidemiology Collaboration (CKD-EPI) equation (16). In accordance with the Kidney Disease Outcomes Quality Initiative (K/DOQI) guidelines, renal impairment was categorized into stages CKD1, CKD2, CKD3, CKD4 and CKD5, based on the eGFR (17). PCT was assessed with the CL-6000i chemiluminescence analyzer (Mindray, China). D-dimer was evaluated using the CS5100 coagulation analyzer (Sysmex, Japan). CRP was assessed with the BN II Special Protein Analyzer (Siemens, Germany).

Regarding the evaluation of autoantibodies, this study encompassed the following components: anti-MPO, anti-PR3, ANAs, anti-double stranded DNA (anti-dsDNA), anti-CCP, as well as anti-glomerular basement membrane (anti-GBM). Anti-MPO and anti-PR3 were identified utilizing the immunoblotting (Kangrun Biotech, China). ANAs and anti-dsDNA were detected through the indirect immunofluorescence method (EUROIMMUN, Germany). Anti-CCP was detected via the chemiluminescence method (HOB, China). Anti-GBM was detected via the chemiluminescence method (Kangrun Biotech, China). Reference ranges for indicators could be found in **Supplementary Table 1**. Quality controls for all tests consistently fell within acceptable ranges daily. All tests were conducted by laboratory professionals who had undergone appropriate training and authorization.

### 2.5 Feature selection and construction of ML analysis

For variable selection, least absolute shrinkage and selection operator (LASSO), logistic regression (LR), and random forest (RF) were used to identify variables with P-values less than 0.05. The intersection of variables was determined to identify the key variables for this study. Additionally, variables not present in the intersection but possessing potential clinical significance were also considered. Following the aggregation of the variables, the final set for ML in this study was established.

This study reviewed existing ML algorithms and selected five models for analysis: LR, RF, support vector classification (SVC), light gradient boosting machine (LGBM) and extreme gradient boosting machine (XGBM). These algorithms had demonstrated clinical applicability in various studies (18–20). For this research, the entire dataset was utilized as the training set, and all models underwent parameter optimization via grid search and 5-fold cross-validation. Receiver operating curves (ROC) were generated, and metrics such as sensitivity, specificity, F1 score, and G-Mean were evaluated to identify the optimal machine learning model. Following the optimal model, SHapley Additive exPlanations (SHAP) was employed to quantify feature importance, assess feature contributions and illustrate nonlinear relationships.

In this study, the R packages utilized for each ML algorithm were as follows: LASSO regression used the glmnet package (version 4.1.8), LR utilized the rms package (version 6.7.1), RF used the randomForest package (version 4.7-1.2), SVC algorithm was facilitated by the e1071 package (version 1.7-16), LGBM algorithm leveraged the lightgbm package (version 4.5.0), and XGBM algorithm was executed using the xgboost package (version 1.7.8.1).

### 2.6 Evaluation of model’s clinical applicability

Leveraging the advantages of the ML model, we synthesized the effects of multiple variables into a single numerical score to evaluate the clinical utility of indicators. The “rms” package (version 6.7.1) facilitated the construction of a nomogram, illustrating the relationship between each variable within the prediction model. We utilized a calibration curve to determine the necessity for model correction. And the decision curve analysis (DCA) was employed to evaluate the model’s clinical applicability. The nomogram was subsequently applied to a validation set, with the C-index used to assess its effectiveness.

### 2.7 Statistical analysis

The data were processed and analyzed utilizing R (version 4.0.3) and Social Sciences software (version 26.0). Clinical characteristics were presented as counts and percentages. The normality of the data distribution was assessed using the Kolmogorov-Smirnov test. For data exhibiting a normal distribution, the mean and standard deviation were used for description, and comparisons between groups were conducted using an independent samples t-test. In cases where the data did not follow a normal distribution, the median and interquartile range were employed for description, and the Wilcoxon rank-sum test was utilized for group comparisons. Categorical data were described as frequencies and percentages, with comparisons between groups performed using the chi-square test. We employed bootstrap with 1000 iterations and a seed of 1234 to check the stability of the area under the curve (AUC), represented by the C-index. Hosmer-Lemeshow Test was used to assess the goodness of fit in binary logistic regression. If a *P* value was above 0.05, it indicated a good fit and reflected the data characteristics well.

## 3 Results

### 3.1 Changes of anti-MPO detection in the past 8 years

**Figure 2:**
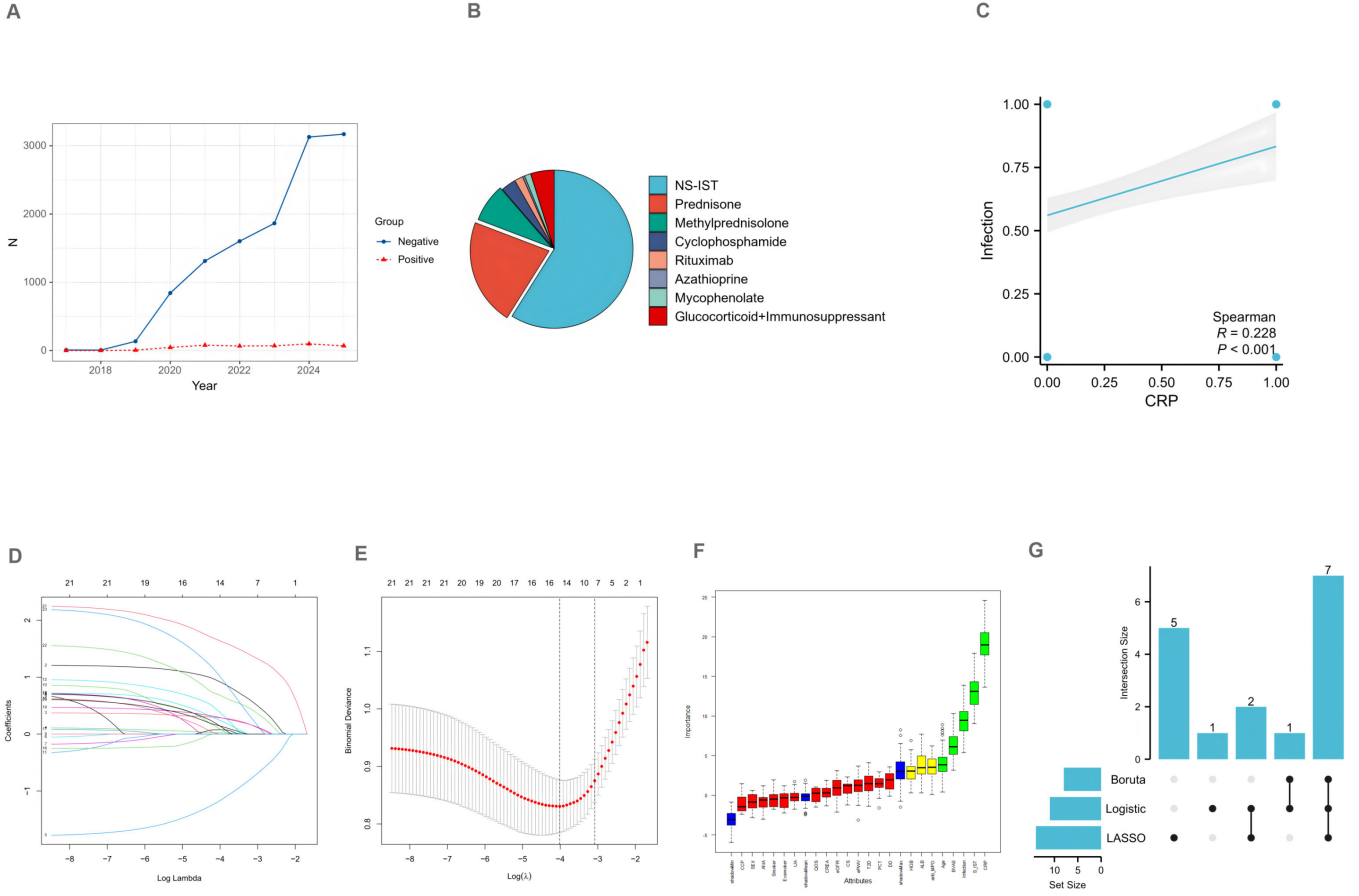
Machine learning algorithms selected for variable screening in this study. **(A)** The trend of 12,497 patients who underwent myeloperoxidase testing over an 8-year period. **(B)** Standardization of immunotherapy among MPA patients. **(C)** A Spearman correlation analysis on infection and CRP. **(D)** LASSO coefficient profiles of the 14 features. A coefficient profile plot was produced against the log(λ). **(E)** The partial likelihood deviance (binomial deviance) curve was plotted versus log (λ). Dotted vertical lines were drawn at the optimal values by using the minimum criteria and the 1 SE of the minimum criteria (the 1-SE criteria). **(F)** The horizontal axis represented attributes of variables, and they were divided into 3 groups based on the shadow value: min, mean and max. The vertical axis represented the importance of each variable. Green represented confirmed, yellow represented tentative, red represented not recommended. **(G)** UpSet diagram was used to display the intersection of the variables of 3 algorithms (LASSO, logistic and Boruta), resulting in a total of 7 variables. LASSO, least absolute shrinkage and selection operator.

Based on the analysis of data from 12,497 patients who underwent anti-MPO testing in Deyang People’s Hospital between November 2017 and October 2025, it was observed that the acceptance of anti-MPO tests by clinical physicians was increasing annually. However, overall positive rate remained relatively stable, as illustrated in **Figure 2A**. Examinations of the number and positive rate of anti-MPO across different age groups revealed that the positive rate varied with age. The positive rate was approximately 1.87% among individuals under 60 years old. And around 3% among individuals aged 61–70 years old. But in individuals aged above 71 years old, positive rate was about 5%. Notably, the number of anti-MPO test submissions from patients over 81 years old was only one-third of that from individuals aged 71-80, indicating a potential risk of missed detection. These findings underscored the necessity of enhancing serological screening for elderly patients presenting with suspicious conditions, as shown in **Table 1**. Among the 230 patients, kidney damage was the most common type, with 155 cases occurring. The next was lung damage, with 140 cases. The incidences skin, eyes, abdomen and cardiovascular system were relatively lower, as shown in **Supplementary Table 2**.

**Table 1:** Changes of myeloperoxidase detection over the past 8 years.

Year of detection	Age (years, positive rate)			
	18-60 (n, %)	61-70 (n, %)	71-80 (n, %)	>80 (n, %)
2017	7 (0)	1 (0)	0 (0)	0 (0)
2018	6 (0)	0 (0)	0 (0)	0 (0)
2019	74 (1.35)	31 (9.68)	23 (4.35)	12 (0)
2020	510 (2.35)	174 (10.92)	151 (5.96)	52 (9.62)
2021	485 (3.92)	433 (6.24)	347 (7.49)	125 (4.80)
2022	858 (1.63)	375 (8.27)	320 (5.31)	115 (3.48)
2023	1006 (1.89)	390 (5.13)	398 (5.53)	139 (5.76)
2024	1652 (1.03)	751 (3.60)	618 (6.80)	205 (5.37)
2025	1638 (0.92)	716 (2.51)	669 (3.74)	216 (4.63)
Mean positive rate	1.87	6.62	5.60	4.81

### 3.2 Characteristics of baseline in MPA hospitalized patients

During the hospitalization period, 56 of 230 MPA patients experienced adverse outcomes. There were five cases who underwent self-discharge, representing 8.9% of poor prognoses MPA patients. On the day of self-discharge, it was documented in the hospital records that the patient’s current BVAS score had not shown improvement. The criteria for determining a critical condition were based on a MEWS score of ≥ 5, indicating that without intensified treatment, the patient’s risk of mortality would substantially increase. Given that there were only 56 cases in the poor prognosis group and the overall number of self-discharged patients was low, a separate analysis of these subgroups was not conducted. Instead, they were collectively categorized under poor prognosis. In evaluating the standardization of immunotherapy among patients, it was observed that 135 individuals either did not utilize immunosuppressants or used them improperly upon admission. In contrast, the remaining 95 patients were undergoing immunosuppressant therapy. Glucocorticoids emerged as the most frequently administered drugs, with Prednisone constituting approximately 21.8% of the total patient cohort, followed by Methylprednisolone at approximately 7.9%. The proportion of patients receiving either immunosuppressants alone or in conjunction with glucocorticoids was below 5% in both cases, as were shown in **Figure 2B** and **Supplementary Table 3**.

A comparative analysis between patients with poor prognosis and those with improved prognosis revealed statistically significant differences in age (χ^2^=13.608, *P* = 0.008), ePWV (χ^2^=8.744, *P* = 0.033), infection (χ^2^=18.013, *P* < 0.001) and S-IST (χ^2^=22.289, *P* < 0.001). No significant differences were observed between the two groups regarding smoking status, including smoker, current smoker, ex-smoker and quantity of smoking (*P*>0.05). Both categorical and continuous variable analyses were performed on the BVAS. Wilcoxon test revealed that the BVAS was higher in the improved group compared to the no-improved group (*Z* = 3.786, *P* = 0.002). Chi-square test indicated that the proportion of non-active patients was lower in the no-improved group (χ^2^=11.341, *P* = 0.003).

Regarding laboratory indicators, patients with poor prognosis exhibited lower levels of HGB (χ^2^=12.032, *P* = 0.007) and albumin (ALB) (χ^2^=12.781, *P* = 0.005), whereas the d-dimer (χ^2^=7.469, *P* = 0.006), CRP (χ^2^=42.843, *P* < 0.001) were higher compared to patients with improved prognosis. The positive rate of anti-MPO was higher in the no-improved group (χ^2^=6.230, *P* = 0.013). There was no significant difference in the positive rates of ANAs, anti-CCP, and anti-GBM between the two groups (*P*>0.05), as detailed in **Table 2**. Results of all indicators were presented in **Supplementary Table 4**. Here we conducted a Spearman correlation analysis on infection and CRP, it was noteworthy that no correlation was exiting, as shown in **Figure 2C**. Therefore, both infection and CRP were included in the subsequent analysis.

**Table 2:** Characteristics of different groups in 230 MPA patients (positive result).

Characteristics	Improved	No-improved	Statistic	*P* value	Method
Age (n, %)			13.608	0.008^*^	Chi-square test
18–60 year	46 (26.4%)	7 (12.5%)			
61–70 year	64 (36.8%)	15 (26.8%)			
71–80 year	51 (29.3%)	24 (42.8%)			
≥81 year	13 (7.5%)	10 (17.9%)			
Estimated pulse wave velocity (n, %)			8.744	0.033^*^	Yates’ correction
≤12.35 m/s	125 (71.8%)	29 (51.8%)			
12.36-13.74 m/s	34 (19.5%)	17 (30.4%)			
13.75-15.16 m/s	11 (6.3%)	6 (10.7%)			
≥15.17 m/s	4 (2.3%)	4 (7.1%)			
S-IST (n, %)			22.289	<0.001^*^	Chi-square test
No	87 (50.0%)	48 (85.7%)			
Yes	87 (50.0%)	8 (14.3%)			
Infection (n, %)			18.013	<0.001*	Chi-square test
No	80 (46.0)	8 (14.3)			
Yes	94 (54.0)	48 (85.7)			
BVAS Score				0.002^*^	Wilcoxon
Median (IQR)	10.0 (2.0, 12.0)	12.0 (7.0, 14.0)			
BVAS Score (n, %)			11.341	0.003^*^	Chi-square test
0≤Score ≤ 5	62 (35.6%)	10 (17.9%)			
6≤Score ≤ 14	100 (57.5%)	35 (62.5%)			
Score≥15	12 (6.9%)	11 (19.6%)			
C-reactive protein (n, %)			42.843	<0.001^*^	Chi-square test
0–10 m/s	155 (89.1%)	27 (48.2%)			
>10	19 (10.9%)	29 (51.8%)			
Hemoglobin (n, %)			12.032	0.007^*^	Yates’ correction
110–160 g/L	57 (32.8%)	14 (25.0%)			
90–109 g/L	51 (29.3%)	9 (16.1%)			
60–89 g/L	63 (36.2%)	28 (50.0%)			
≤59 g/L	3 (1.7%)	5 (8.9%)			
Albumin (n, %)			12.871	0.005^*^	Yates’ correction
>35 g/L	89 (51.1%)	20 (35.7%)			
31–35 g/L	55 (31.6%)	14 (25.0%)			
26–30 g/L	23 (13.2%)	19 (33.9%)			
21–25 g/L	7 (4.0%)	3 (5.4%)			
D-dimer (n, %)			7.469	0.006^*^	Chi-square test
0-0.5 mg/L	50 (28.7%)	6 (10.7%)			
>0.5 mg/L	124 (71.3%)	50 (89.3%)			
Anti-MPO (n, %)			6.230	0.013^*^	Chi-square test
Negative	29 (16.7%)	2 (3.6%)			
Positive	145 (83.3%)	54 (96.4%)			

MPA, microscopic polyarteritis; SI, smoking index, IQR, Interquartile range; BVAS, Birmingham vasculitis activity score; MPO, myeloperoxidase; S-IST, standardized immunosuppressive therapy. ^*^P < 0.05.

### 3.3 Results of ML variable selection

In this study, three algorithms (LASSO, LR and RF) were employed to screen research indicators. Initially, numerical variables were transformed into categorical variables. LASSO regression was first performed with the occurrence of adverse outcomes in patients as the dependent variable. The cross-validation curve analysis identified 14 indicators that corresponded to optimal curve fitting: infection, age, ePWV, S-IST, smoker, exsmoker, ANA, T2D, PCT, albumin, d-dimer, CRP, BVAS, anti-MPO, as illustrated in **Figures 2D, E**. Subsequently, variables with *P* < 0.05 in the LR regression were identified, resulting in the selection of 11 variables: S-IST, CRP, infection, albumin, d-dimer, BVAS, age, HGB, anti-MPO, creatinine and ePWV, as detailed in **Supplementary Table 5**.The RF algorithm was utilized to assess the importance of each variable, retaining those classified as confirmed and tentative, including CRP, S-IST, infection, age, albumin, HGB, anti-MPO and BVAS, as depicted in **Figure 2F**. Seven variables were obtained through the three algorithms, including S-IST, age, CRP, infection, BVAS, albumin and anti-MPO, as shown in **Figure 2G**. Finally, this study included the above 7 variables for ML. In this study, the sample size calculation for binary variables was conducted using the formula referenced in source number (21). The overall outcome proportion was determined to be 6.62% at its maximum level in this study, as detailed in **Supplementary Table 1**. Three ML algorithms identified 7 common factors. Based on these calculations, a minimum of 162 patients with MPA was required for inclusion. Notably, the actual number of MPA patients included in this study was 230, thereby satisfying the sample size requirement prior to the construction of the model.

### 3.4 Evaluate the model using five ML algorithms

**Figure 3:**
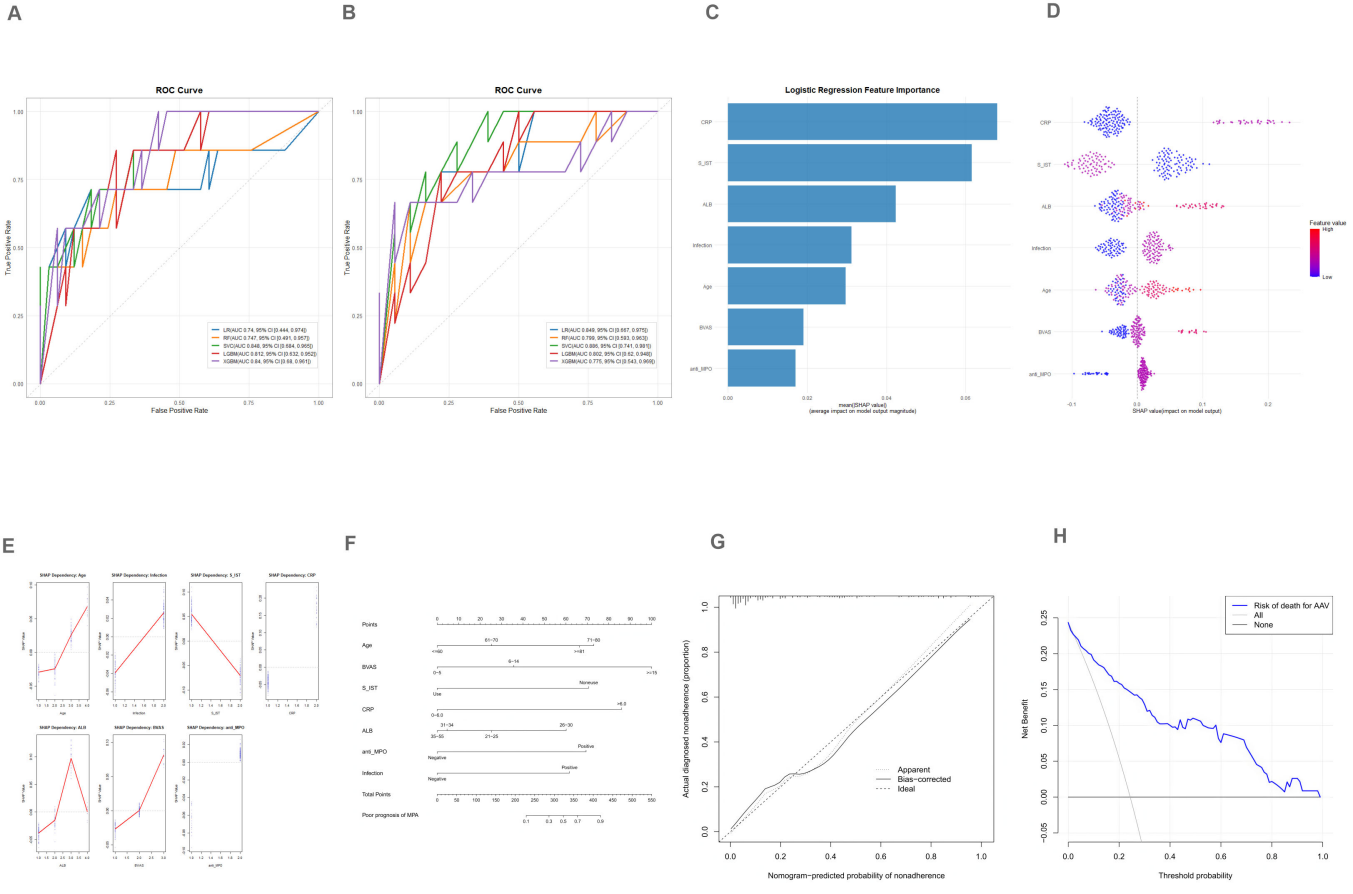
Evaluate the performance of machine learning algorithms and the clinical applicability. **(A)** ROC curves of the prediction models constructed by 5 machine learning algorithms. **(B)** ROC curves of the validation models constructed by 5 machine learning algorithms. **(C)** The SHAP values of 7 variables in the SVC algorithm. The x-axis represented the average impact on the magnitude of the model output, and the y-axis represented the 6 variables in this study. **(D)** SHAP visualization analysis under the SVC algorithm. The x-axis represented the classification situation of each variable, and the y-axis represented the 7 variables. **(E)** SHAP dependence diagrams for the 7 indicators under the SVC algorithm. **(F)** Developing a nomogram for poor prognosis in MPA patients. **(G)** Calibration curve of the nomogram. The *x*-axis represented predicted risk. The *y*-axis represented actual risk. The diagonal dotted line represented a perfect prediction by an ideal model. The solid line represented corrected performance of the nomogram, of which a closer fit to the diagonal dotted line represented a better prediction. **(H)** Decision curve of the nomogram. The *x*-axis represented threshold probability, and the *y*-axis represented net benefit. The horizontal solid line indicated that all patients were negative. The oblique solid line indicated that all patients were positive. ROC, receiver operating curves; SHAP, SHapley Additive exPlanations; SVC, support vector classification; MPA, microscopic polyangiitis.

Utilizing the above 7 indicators as feature variables, five ML algorithms were used to develop a predictive model for adverse prognosis in MPA. Among the algorithms, the SVC exhibited the highest AUC of 0.848, followed by XGBM with an AUC of 0.840, as depicted in **Figure 3A**. All five algorithms demonstrated moderate predictive capabilities. The performance metrics were evaluated, revealing that the F1 score of SVC was 0.600, and the G-Mean was 0.655. In the validation set, the SVC achieved the highest AUC of 0.886, followed by LR, as illustrated in **Figure 3B**. LR exhibited the highest F1 score and G-Mean, as detailed in **Supplementary Table 6**. Additionally, we computed the difference in the AUC between the prediction model and the validation model. The analysis revealed that the differences between the two algorithms were less than 5%. This finding suggested that the LGBM and SVC models did not exhibit overfitting and demonstrated robust generalizability. Based on these results, SVC algorithms demonstrated superior performance and were considered the optimal algorithms. To assess the stability of the model, we conducted Hosmer-Lemeshow Test on 7 variables. The results indicated that there were no significant differences among each step, suggesting that the prediction model fitted well and reflected the data, as shown in **Supplementary Table 7**.

### 3.5 Interpretability of the SVC algorithms

Utilizing SHAP for importance analysis, we evaluated the significance of 7 indicators, presented in **Figure 3C**. In SVC algorithm, the most influential factor was CRP, followed by whether the patient received S-IST and moderate serum albumin reduction. To elucidate the decision-making processes of the SVC algorithm, we conducted a visual analysis of SHAP. Firstly, an elevated CRP level significantly increased the risk of adverse outcomes when patients were admitted to the hospital. Secondly, patients undergoing immunosuppressive therapy demonstrated a significantly reduced likelihood of experiencing adverse outcomes. Thirdly, patients with albumin between 25–30 g/L were at a heightened risk of adverse outcomes. In addition, the presence of infection upon admission, age greater than 71 years, a BVAS greater than 15, and positive anti-MPO antibodies were all associated with a poor prognosis for the MPA patients, as illustrated in **Figure 3D**. Finally, we calculated the SHAP dependence diagrams for the 7 indicators. The distribution patterns of the 7 indicators under SVC algorithm were basically the same. Among them, S-IST reduced the risk of adverse outcomes. Age, infection and BVAS showed significant stratification changes, as shown in **Figure 3E**.

### 3.6 Results of nomograms and clinical applicability evaluation

We used a nomogram to assess the contribution of 7 indicators within a unified scoring framework. The nomogram revealed that increased CRP, non-standardized use of immunosuppressants, advanced age and a BVAS greater than 15 were associated with high individual scores as illustrated in **Figure 3F**. The C-index was 0.891, suggesting that the nomogram model possessed strong discriminatory capability, as detailed in **Supplementary Table 8**. The calibration curve analysis indicated that the calibration curve of this model aligned with the trend post-bias correction, exhibiting no significant bias, thus obviating the need for further bias correction, as depicted in **Figure 3G**. DCA curve indicated that the curve was above the two reference lines of “treat none and treat all”, and had high practical value in clinical decision-making, as shown in **Figure 3H**. To evaluate the relationship between the size of the validation set and the 95% confidence interval (CI), we computed AUC and 95% CI for various validation sets, determined by different splitting ratios. The findings indicated that as the number of samples in the validation set increased, the AUC values consistently demonstrated a moderate predictive capability, while the 95% CI remained relatively stable. These results suggested that in cases with limited sample sizes, it was crucial to calculate the AUC across different splitting ratios to prevent instability in the AUC and the expansion of CIs. For further details, refer to **Supplementary Table 9**.

## 4 Discussion

This study represented the first single-center, retrospective cohort analysis of patients with MPA in the southwestern of China, spanning an 8-year period. The findings indicated that the frequency was higher among individuals aged 61-80 but decreased in those over 81 years old. Regarding the positive rate of anti-MPO, it was observed to be higher in individuals over 71 years old and remained stable. Epidemiological studies suggested that the incidence of MPA increased significantly with age. During the 1980s, the peak age was between 65–74 years (22–24). By 2007-2013, the peak age of diagnosis in the same region had shifted to 84 years and older (25). Concerning the incidence of AAV subtypes, the age of diagnosis for MPA was higher compared to granulomatosis with GPA and was associated with a poorer prognosis (26–28). In this study, we observed that while the positive rates of anti-MPO were comparable between the 71–80 group and those aged 81 and above, the number of cases submitted for testing in the 81 and above group was only one-third of that in the former. It was suggested a potential risk of under-detection. At the same time, the baseline data at the time of admission revealed that individuals aged over 71 years demonstrated a positive correlation with the incidence of adverse outcomes. Advanced age demonstrated a positive correlation with poor prognosis, corroborating recent epidemiological findings (29). These studies indicated that enhancing screening and early intervention for MPA patients, particularly among the elderly, was important for improving the prognosis.

There was no consensus regarding the regional and population-based differences in the incidence and clinical manifestations of vasculitis. Initial studies investigating granulomatosis with polyangiitis among European white and African populations indicated that Black patients exhibited a higher likelihood of severe granuloma symptoms and experienced significantly shorter recurrence intervals (25). Conversely, research examining the incidence of AAV within a mixed-race population in the United Kingdom revealed that the crude incidence rate among Black and Minority Ethnic (BME) groups was lower compared to that of the white population (30). Currently, the differences in MPA among various ethnic groups are still under investigation. This study focused on the Han ethnicity in Southwest China, which made up over 95% of the population in this region, providing broad reference value.

The pathogenesis of MPA remained an active area of investigation. ANCA played a critical role by activating neutrophils, which adhered to the endothelial cells of small blood vessels. This interaction was considered the initial trigger for MPA (31). Upon abnormal activation, neutrophils released MPO proteases, disrupting immune tolerance and promoting the production of ANCA. The binding of ANCA to the MPO antigen on the surface of neutrophils led to the release of neutrophil extracellular traps (NETs) (32). NETs exacerbated endothelial cell damage and inflammatory responses and also activated the complement alternative pathway (33). Neutrophils activated by the complement system, along with monocytes and macrophages, sustain inflammation and contribute to organ damage (34, 35). Over the past two decades, the challenge of inhibiting the overactive immune system has been a significant focus for clinicians. The induction strategies for MPA remission have transitioned from relying on cyclophosphamide to rituximab, azathioprine and others. Clinicians were investigating approaches to shorten the duration of glucocorticoid therapy and to reduce or discontinue glucocorticoid use (36, 37). In this study, we observed that patients who received standard immunosuppressive therapy exhibited a downward trend on poor prognosis for MPA. The nomogram model indicated that the score of standardized use of immunosuppressants contributed to the likelihood of poor prognosis in MPA. This finding underscored the necessity for clinicians to pay attention. Recurrence remained a significant challenge in maintaining remission. Despite efforts to substantially decrease the reliance on glucocorticoids, prior to the adoption of novel immunomodulatory treatment strategies, it was imperative for clinicians to enhance educational initiatives for MPA patients who were undergoing or preparing to undergo immunosuppressive therapy. Based on this study, the implementation of standardized monitoring and follow-up protocols for immunosuppressive therapy was crucial for disease management.

To ensure that the results of rare diseases were not unstable due to the small sample size, we conducted the following analyses based on literatures. To mitigate overfitting, we employed a time-based verification by organizing patients chronologically according to their admission dates. Following a 7:3 split ratio, the patients were allocated into two distinct datasets: a training set and a validation set. This method had been proven in numerous studies to be the most reliable analytical method when there was no external validation (38). When selecting variables, we removed those that were not related to MPA. We used the LASSO for dimensionality reduction, effectively controlling the number of variables. Secondly, in terms of the variables in ML, we used three widely recognized selection methods and examined the intersection of the features. Despite the differing feature selection techniques, the resulting feature variables demonstrated consistency. Thirdly, we used the calculation formula for small sample size in binary classification in the sample size assessment. The calculated minimum sample size was 162 cases, which was less than the 230 cases included in this study. This indicated that the sample size met the minimum standard for ML. In the context of constructing ML models, we developed five models in current research and identified the optimal model using metrics such as the ROC curve, F1 score, and G-mean (19, 39, 40). Fourthly, in order to assess the stability of the prediction model, we conducted Hosmer-Lemeshow Test on 7 variables. The results indicated that there were no significant differences in each step, suggesting that the prediction model fitted well and reflected the overall data. Furthermore, we calculated the difference in AUC between the prediction model and the validation model. The result showed that the difference between the prediction model and the validation model was less than 5%. This finding indicated that the SVC model did not exhibit overfitting and had good generalization ability. Finally, we evaluated the clinical value and practicability. SHAP dependency graphs revealed that the distribution patterns of the 7 indicators across different algorithms were largely similar. Calibration curve and the DCA supported that this nomogram had significant clinical value. These findings suggested that the predictive model offered reference value for clinicians in assessing the prognosis of MPA.

The calculation of sample size is based on the two-sided confidence interval for single-sample sensitivity and specificity analysis (41). As machine learning models continue to evolve, an increasing number of researchers are striving to develop methods for estimating or calculating the minimum sample size required for diagnostic studies (42, 43). It is important to note that there is currently no universally superior technique, as the choice of method is contingent upon the specific research objectives and the expectations of the researchers. In the context of modeling common diseases, employing large sample sizes facilitates the attainment of narrower 95% confidence intervals, thereby enhancing the precision of machine learning outcomes. Some scholars advocate that, in the development of large predictive models, establishing a narrower expected width (e.g., 0.1-0.2) for the 95% confidence interval can be instrumental in emphasizing predictive efficacy (44, 45). However, when addressing rare diseases and small sample single-center data, the question arises: how should the diagnostic efficacy of the model be evaluated? Previous studies had been corroborated using public databases (46). However, the AAV clinical information from public databases concerning was relatively scarce. In this study, we concentrated on evaluating four aspects: the selection of predictive factors, model performance assessment, internal validation, and the evaluation of the model’s applicability in clinical settings. Nonetheless, we identified that for rare diseases, the reliance on single-center small sample sizes presented a significant data bottleneck. For instance, when we partitioned the overall dataset at varying proportions, the 95% CIs remained wide. We aspire to develop a free online mini-program to attract more researchers to utilize our model. Additionally, we hope to engage in multi-center research collaborations to refine the model’s inclusion criteria, ultimately striving to develop a real-time analysis program that garners acceptance from clinicians.

Early investigations have identified infection and active vasculitis as significant contributors to early mortality in patients with AAV (47). Notably, infections may be associated with short-term high-dose immunosuppressive therapy (48). Clinical research indicates that patients undergoing cyclophosphamide induction therapy exhibit a higher propensity for acute infections compared to those treated with rituximab and methotrexate (49). The etiology of these infections is multifaceted, involving various pathogens, and traditional pathogenic microorganism tests alone are inadequate. In this study, we extracted infection diagnoses from the hospital admission index page of patients, encompassing bacteria, fungi, viruses, and other pathogens, rather than relying solely on results from pathogenic microorganism cultures. In this study, we incorporated both CRP and infection variables concurrently into the machine learning model. Our analysis of the correlation between infection and CRP revealed no significant association between these variables in patients with MPA. Given that CRP is induced by the response to inflammatory cytokines, particularly interleukin-6 (IL-6), it serves as a reliable marker of systemic immune activation (50). We posit that in MPA patients, elevated CRP levels indicate a sustained pro-inflammatory state. In a prospective observational study, elevated CRP levels in critically ill patients were correlated with a heightened risk of re-hospitalization (51). A significant proportion of these patients experience ongoing inflammation, which has been demonstrated to be associated with adverse prognoses. The authors posited that increased CRP levels were indicative of the persistent pro-inflammatory state in critically ill patients. Furthermore, a sustained chronic inflammatory state, even at subclinical levels, was linked to unfavorable long-term outcomes (52). In a recent investigation, it was determined that CRP served as a reliable marker for evaluating the inflammatory state and mortality risk in individuals infected with the novel coronavirus (SARS-CoV-2) (53). Another study assessed COVID-19 patients using the modified early warning score (MEWS) and concluded that CRP increased with disease severity, making it a valuable indicator for distinguishing between mild and severe COVID-19 cases and predicting mortality risk (54). In our study, we observed that among patients with poor prognosis in MPA, elevated CRP levels at admission were positively correlated with adverse outcomes, aligning with the aforementioned studies. We proposed that CRP was a valuable marker for indicating the body’s chronic inflammatory condition.

While the recognition of hypoalbuminemia as a poor prognostic indicator in patients was not unprecedented, and numerous studies had established the association with increased mortality in diseases (55, 56). This study was the first to examine the relationship between hypoalbuminemia at the time of admission and poor prognosis in MPA. Utilizing established criteria for hypoalbuminemia, we categorized patients’ albumin into mild (3.0-3.5 g/dL), moderate (2.5-3.0 g/dL), and severe (<2.5 g/dL) groups. Our analysis revealed that patients presenting with moderate hypoalbuminemia at admission exhibited the highest risk of adverse outcomes. This finding contrasted with previous studies that had typically reported a dose-dependent relationship between lower albumin levels and increased mortality (57–59). This phenomenon was attributable to two factors. First, there were 10 patients exhibiting severe albumin reduction within this study. Among these, seven experienced a favorable prognosis while three had a poor prognosis. The limited sample size could introduce bias into the ML algorithm. Second, variations in the classification of albumin reduction in different studies contributed to discrepancies in findings. For instance, some studies categorized albumin reduction as mild (<3.5 g/dL) or severe (<2.5 g/dL), potentially leading to negative results. Given that MPA is a rare disease, we hope to conduct multi-center validation in the future.

This study is subject to several limitations. Firstly, as a single-center retrospective analysis, despite repeated evaluations of result stability at each analytical stage, future research necessitates the use of multi-center data to validate its applicability in external contexts. Secondly, the primary organs affected in the patient cohort were the lungs and kidneys. The study does not adequately address early risk assessment for gastrointestinal damage caused by MPA, particularly in severe cases such as organ perforation, which remains a critical area for ongoing investigation. Thirdly, the clinical condition of MPA patients during hospitalization is subject to dynamic changes. Although baseline data at admission were utilized for predictive purposes, the study did not account for changes in dynamic indicators over time. Consequently, we are currently unable to ascertain the value of the test indicators included in the model for dynamic monitoring. Future research should focus on multi-center validation, further optimization of potential prognostic markers, and the development of a freely accessible online platform for clinicians to enhance reference utility.

In conclusion, we developed a prediction model for adverse outcomes in MPA patients, utilizing clinical and laboratory data collected on the day of admission. SVC algorithm exhibited moderate predictive efficacy. Factors such as elevated serum CRP levels, moderate reductions in serum albumin, infection, advanced age, BVAS larger than 15, and anti-MPO were positively associated with adverse prognoses. Standardized immunosuppressive therapy was shown to mitigate this risk, offering a valuable reference for clinical decision-making.

## Data Availability

The original contributions presented in the study are included in the article/**Supplementary Material**. Further inquiries can be directed to the corresponding author.
