# Supplementary material for: Developing a prediction model for poor prognosis in MPA patients using initial admission examination results: a machine learning study from Southwest China
Source: Front Immunol. 2026 Jul 1;17:1820073. doi: 10.3389/fimmu.2026.1820073 (PMC13369057; doi:10.3389/fimmu.2026.1820073)
Supplement: Supplementary file 1 [file SupplementaryFile1.docx]

**Supplementary Table 1. The reference value range for the included indicators**

| **Variable** | **Method** | **Reference range** | **Unit** |
| --- | --- | --- | --- |
| Procalcitonin | CLIA | 0-0.05 | ng/ml |
| C-reactive protein | Nephelometry | 0-6 | mg/L |
| D-dimer | Immunoturbidimetry | 0.08-0.55 | mg/L |
| Total protein | Biuret method | 65-85 | g/L |
| Albumin | Bromocresol green method | 40-55 | g/L |
| Serum creatinine | Sarcosine oxidase method | 41-81 (female)/57-111 (male) | umol/L |
| Urea | Urease method | 3.1-8.80 (female)/3.6-9.6 (male) | mmol/L |
| Uric acid | Uricase method | 155-357 (female)/208-428 (male) | umol/L |
| Cystatin C | Immunoturbidimetry | 0.64-1.23 | mg/L |
| Anti-MPO | Immunoblotting | Negative |  |
| Anti-PR3 | Immunoblotting | Negative |  |
| ANAs | IFA | <1:80 |  |
| Anti-dsDNA | IFA | Negative |  |
| Anti-CCP | CL | 0-20 | RU/ml |
| Anti-GBM | CL | 0-20 | U/ml |

Anti-MPO, anti-myeloperoxidase; anti-PR3, anti-proteinase 3; ANAs, antinuclear antibodies; anti-CCP, anti-cyclic citrullinated peptide; anti-GBM, anti-glomerular basement membrane; CLIA, chemiluminescent immunoassay; IFA, indirect immunofluorescence assay; CL, chemiluminescence method.

**Supplementary Table 2. Proportion of organ damage in 230 MPA patients**

| **Organ damage** | **Number** | **Percentage (%)** |
| --- | --- | --- |
| Systematicness | 16 | 6.96 |
| Skin | 4 | 1.74 |
| Mucosa/eye | 2 | 0.87 |
| Ear-nose-throat | 3 | 1.30 |
| Chest | 140 | 60.87 |
| Cardiovascular | 1 | 0.43 |
| Abdomen | 1 | 0.43 |
| Kidney | 155 | 67.39 |
| Nervous system | 7 | 3.04 |

MPA, microscopic polyangiitis.

**Supplementary Table 3. Medication usage of 230 MPA patients upon admission**

| **Medication usage** | **Number** | **Percentage (%)** |
| --- | --- | --- |
| NS-IST | 135 | 58.7 |
| Prednisone | 50 | 21.8 |
| Methylprednisolone | 18 | 7.9 |
| Cyclophosphamide | 7 | 3.1 |
| Rituximab | 4 | 1.8 |
| Azathioprine | 1 | 0.4 |
| Mycophenolate | 3 | 1.4 |
| Glucocorticoid+Immunosuppressant | 11 | 4.9 |

MPA, microscopic polyangiitis; NS-IST, non-standardized immunosuppressive therapy.

**Supplementary Table 4. Characteristics of different groups in 230 microscopic polyarteritis patients**

| **Characteristics** | **Improved** | **No-improved** | **Statistic** | **P value** | **Method** |
| --- | --- | --- | --- | --- | --- |
| Age (n, %) |  |  | 13.608 | 0.008* | Chi-square test |
| 18-60 year | 46 (20.0%) | 7 (3.1%) |  |  |  |
| 61-70 year | 64 (27.8%) | 15 (6.5%) |  |  |  |
| 71-80 year | 51 (22.2%) | 24 (10.4%) |  |  |  |
| ≥81 year | 13 (5.7%) | 10 (4.3%) |  |  |  |
| Sex (n, %) |  |  | 0.475 | 0.491 | Chi-square test |
| Male | 90 (39.1%) | 26 (11.3%) |  |  |  |
| Female | 84 (36.5%) | 30 (13%) |  |  |  |
| Type 2 diabetes (n, %) |  |  | 4.770 | 0.092 | Yates' correction |
| No | 144 (62.6%) | 46 (20%) |  |  |  |
| Without complications | 27 (11.7%) | 6 (2.6%) |  |  |  |
| With complications | 3 (1.3%) | 4 (1.7%) |  |  |  |
| Hypertension (n, %) |  |  | 1.335 | 0.721 | Yates' correction |
| No | 91 (39.6%) | 25 (10.9%) |  |  |  |
| Grade 1 | 63 (27.4%) | 22 (9.6%) |  |  |  |
| Grade 2 | 15 (6.5%) | 7 (3%) |  |  |  |
| Grade 3 and 4 | 5 (2.2%) | 2 (0.9%) |  |  |  |
| Estimated pulse wave velocity (n, %) | |  | 8.744 | 0.033^*^ | Yates' correction |
| ≤12.35 m/s | 125 (54.3%) | 29 (12.6%) |  |  |  |
| 12.36-13.74 m/s | 34 (14.8%) | 17 (7.4%) |  |  |  |
| 13.75-15.16 m/s | 11 (4.8%) | 6 (2.6%) |  |  |  |
| ≥15.17 m/s | 4 (1.7%) | 4 (1.7%) |  |  |  |
| Standardized immunosuppressive therapy (n, %) | | | 22.289 | <0.001^*^ | Chi-square test |
| No | 87 (37.8%) | 48 (20.9%) |  |  |  |
| Yes | 87 (37.8%) | 8 (3.5%) |  |  |  |
| Smoker (n, %) |  |  |  |  |  |
| No | 124 (53.9%) | 35 (15.2%) | 1.525 | 0.217 | Chi-square test |
| Yes | 50 (21.7%) | 21 (9.1%) |  |  |  |
| Current Smoker (n, %) | |  | 1.689 | 0.194 | Chi-square test |
| No | 152 (66.1%) | 45 (19.6%) |  |  |  |
| Yes | 22 (9.6%) | 11 (4.8%) |  |  |  |
| Ex-smoker (n, %) |  |  | 0.096 | 0.757 | Chi-square test |
| No | 146 (63.5%) | 46 (20.0%) |  |  |  |
| Yes | 28 (12.2%) | 10 (4.3%) |  |  |  |
| Quantity of smoking (SI) | | | 2.291 | 0.514 | Yates' correction |
| 0 | 124 (53.9%) | 35 (15.2%) |  |  |  |
| 0-200 | 7 (3.0%) | 3 (1.3%) |  |  |  |
| 201-400 | 4 (1.7%) | 3 (1.3%) |  |  |  |
| ≥400 | 39 (17.0%) | 15 (6.5%) |  |  |  |
| BVAS Score | | | 3.786 | 0.002^*^ | Wilcoxon |
| Median (IQR) | 10.0 (2.0, 12.0) | 12.0 (7.0, 14.0) |  |  |  |
| BVAS Score (n, %) |  |  | 11.341 | 0.003^*^ | Chi-square test |
| 0-5 | 62 (27.0%) | 10 (4.3%) |  |  |  |
| 6-14 |  |  |  |  |  |
| ≥15 |  |  |  |  |  |
| Infection (n, %) |  |  | 18.013 | <0.001^*^ | Chi-square test |
| No | 80 (46.0) | 8 (14.3) |  |  |  |
| Yes | 94 (54.0) | 48 (85.7) |  |  |  |
| C-reactive protein (n, %) | |  | 42.843 | <0.001^*^ | Chi-square test |
| 0-10 m/s | 155 (67.4%) | 27 (11.7%) |  |  |  |
| >10 | 19 (8.3%) | 29 (12.6%) |  |  |  |
| Blood urea nitrogen (n, %) | |  | 1.255 | 0.264 | Chi-square test |
| 2.9-7.5 mmol/L | 54 (23.5%) | 13 (5.7%) |  |  |  |
| Abnormal | 120 (52.2%) | 43 (18.7%) |  |  |  |
| Uric acid (n, %) |  |  | 3.134 | 0.077 | Chi-square test |
| 208-428 (male)/155-357 umol/L (female) | 116 (50.4%) | 30 (13%) |  |  |  |
| Abnormal | 58 (25.2%) | 26 (11.3%) |  |  |  |
| Creatinine (n, %) |  |  | 7.581 | 0.055 | Chi-square test |
| 0-133 mmol/L | 73 (31.7%) | 19 (8.3%) |  |  |  |
| 134-177 mmol/L | 34 (14.8%) | 6 (2.6%) |  |  |  |
| 178-443 mmol/L | 29 (12.6%) | 18 (7.8%) |  |  |  |
| >443 mmol/L | 38 (16.5%) | 13 (5.7%) |  |  |  |
| Cystatin C (n, %) |  |  | 0.044 | 0.834 | Chi-square test |
| 0.51-1.09 mg/L | 32 (13.9%) | 11 (4.8%) |  |  |  |
| Abnormal | 142 (61.7%) | 45 (19.6%) |  |  |  |
| White blood cell (n, %) | |  | 1.566 | 0.457 | Yates' correction |
| 3.5-9.5×10^9^/L | 100 (43.5%) | 35 (15.2%) |  |  |  |
| <3.5×10^9^/L | 5 (2.2%) | 3 (1.3%) |  |  |  |
| >9.5×10^9^/L | 69 (30%) | 18 (7.8%) |  |  |  |
| Absolute neutrophil count (n, %) | |  | 1.378 | 0.240 | Chi-square test |
| <6.3×10^9^/L | 90 (39.1%) | 34 (14.8%) |  |  |  |
| >6.4×10^9^/L | 84 (36.5%) | 22 (9.6%) |  |  |  |
| Absolute monocyte count (n, %) | |  | 0.004 | 0.947 | Chi-square test |
| ≤0.6×10^9^/L | 111 (48.3%) | 36 (15.7%) |  |  |  |
| >0.6×10^9^/L | 63 (27.4%) | 20 (8.7%) |  |  |  |
| Absolute lymphocyte count (n, %) | |  | 0.825 | 0.662 | Yates' correction |
| 1.1-3.2×10^9^/L | 83 (36.1%) | 23 (10%) |  |  |  |
| <1.1×10^9^/L | 89 (38.7%) | 32 (13.9%) |  |  |  |
| >3.2×10^9^/L | 2 (0.9%) | 1 (0.4%) |  |  |  |
| Hemoglobin (n, %) |  |  | 12.032 | 0.007^*^ | Yates' correction |
| 110-160 g/L | 57 (24.8%) | 14 (6.1%) |  |  |  |
| 90-109 g/L | 51 (22.2%) | 9 (3.9%) |  |  |  |
| 60-89 g/L | 63 (27.4%) | 28 (12.2%) |  |  |  |
| ≤59 g/L | 3 (1.3%) | 5 (2.2%) |  |  |  |
| Platelet (n, %) |  |  | 1.647 | 0.439 | Yates' correction |
| 70-300×10^12^/L | 125 (54.3%) | 44 (19.1%) |  |  |  |
| <70×10^12^/L | 14 (6.1%) | 5 (2.2%) |  |  |  |
| >300×10^12^/L | 35 (15.2%) | 7 (3%) |  |  |  |
| Procalcitonin (n, %) |  |  | 2.851 | 0.091 | Chi-square test |
| 0-0.25 ng/ml | 115 (50%) | 30 (13%) |  |  |  |
| >0.25 ng/ml | 59 (25.7%) | 26 (11.3%) |  |  |  |
| Albumin (n, %) |  |  | 12.871 | 0.005^*^ | Yates' correction |
| >35 g/L | 89 (38.7%) | 20 (8.7%) |  |  |  |
| 31-35 g/L | 55 (23.9%) | 14 (6.1%) |  |  |  |
| 26-30 g/L | 23 (10%) | 19 (8.3%) |  |  |  |
| 21-25 g/L | 7 (3%) | 3 (1.3%) |  |  |  |
| D-dimer (n, %) |  |  | 7.469 | 0.006^*^ | Chi-square test |
| 0-0.5 mg/L | 50 (21.7%) | 6 (2.6%) |  |  |  |
| >0.5 mg/L | 124 (53.9%) | 50 (21.7%) |  |  |  |
| Anti-MPO (n, %) |  |  | 6.230 | 0.013^*^ | Chi-square test |
| Negative | 29 (12.6%) | 2 (0.9%) |  |  |  |
| Positive | 145 (63.0%) | 54 (23.5%) |  |  |  |
| Anti-GBM (n, %) |  |  | <0.001 | 1.000 | Yates' correction |
| Negative | 172 (74.8%) | 56 (24.3%) |  |  |  |
| Positive | 2 (0.9%) | 0 (0.0%) |  |  |  |
| ANAs (n, %) |  |  | 0.806 | 0.369 | Chi-square test |
| Titer≤1:100 | 154 (67.0%) | 47 (20.4%) |  |  |  |
| Titer≥1:320 | 20 (8.7%) | 9 (3.9%) |  |  |  |
| Anti-CCP (n, %) |  |  | <0.001 | 1.000 | Yates' correction |
| Negative | 167 (72.6%) | 54 (23.5%) |  |  |  |
| Positive | 7 (3.0%) | 2 (0.9%) |  |  |  |
| Anti-dsDNA (n, %) |  |  | - | - | - |
| Negative | 174 (75.6%) | 56 (24.4%) |  |  |  |
| Positive | 0 | 0 |  |  |  |
| Anti-PR3 (n, %) |  |  | - | - | - |
| Negative | 174 (75.6%) | 56 (24.4%) |  |  |  |
| Positive | 0 | 0 |  |  |  |

^*^*P*<0.05. SI, smoking index, IQR, Interquartile range; BVAS, Birmingham vasculitis activity score (BVAS); MPO, myeloperoxidase; S-IST, standardized immunosuppressive therapy.

**Supplementary Table 5. Variables with a *P*-value less than 0.05 through logistic regression analysis**

| **Variable** | **Estimate** | **Standard error** | **Statistic** | **Odds ratio** | ***P* value** |
| --- | --- | --- | --- | --- | --- |
| CRP | 2.170 | 0.361 | 6.010 | 8.760 | <0.001 |
| S-IST | -1.790 | 0.411 | -4.360 | 0.167 | <0.001 |
| Infection | 1.630 | 0.411 | 3.970 | 2.280 | <0.001 |
| Albumin | 1.300 | 0.397 | 3.280 | 1.690 | 0.001 |
| BVAS | 1.740 | 0.539 | 3.220 | 1.980 | 0.001 |
| Age | 1.620 | 0.584 | 2.770 | 1.610 | 0.006 |
| D-dimer | 1.210 | 0.463 | 2.620 | 1.350 | 0.009 |
| HGB | 1.910 | 0.789 | 2.427 | 1.450 | 0.015 |
| Anti-MPO | 1.690 | 0.748 | 2.250 | 1.250 | 0.024 |
| Creatinine | 0.869 | 0.395 | 2.200 | 1.100 | 0.028 |
| Estimated pulse wave velocity | 0.775 | 0.393 | 1.970 | 1.000 | 0.049 |

CRP, C-reactive protein; S-IST, standardized immunosuppressive therapy; BVAS, Birmingham vasculitis activity score; HGB, hemoglobin.

**Supplementary Table 6. Evaluation of the efficacy of the prediction model and the validation model**

| **Model** | **AUC** | **Accuracy** | **Sensitivity** | **Specificity** | **F1 Score** | **G mean** |
| --- | --- | --- | --- | --- | --- | --- |
| Prediction model |  |  |  |  |  |  |
| LR | 0.740 | 0.900 | 0.429 | 1.000 | 0.600 | 0.655 |
| Random forest | 0.747 | 0.875 | 0.286 | 1.000 | 0.444 | 0.535 |
| SVC | 0.848 | 0.900 | 0.429 | 1.000 | 0.600 | 0.655 |
| LGBM | 0.812 | 0.850 | 0.143 | 1.000 | 0.250 | 0.378 |
| XGBM | 0.840 | 0.825 | 0.286 | 0.939 | 0.364 | 0.518 |
| Validation model |  |  |  |  |  |  |
| LR | 0.849 | 0.778 | 0.667 | 0.833 | 0.667 | 0.745 |
| Random forest | 0.799 | 0.741 | 0.444 | 0.889 | 0.533 | 0.629 |
| SVC | 0.886 | 0.778 | 0.444 | 0.944 | 0.571 | 0.648 |
| LGBM | 0.802 | 0.741 | 0.444 | 0.889 | 0.533 | 0.629 |
| XGBM | 0.775 | 0.704 | 0.667 | 0.722 | 0.600 | 0.694 |
| (AUCp-AUCv)/AUCp | |  |  |  |  |  |
| LR | 0.147 |  |  |  |  |  |
| Random forest | 0.070 |  |  |  |  |  |
| SVC | 0.045 |  |  |  |  |  |
| LGBM | 0.012 |  |  |  |  |  |
| XGBM | 0.077 |  |  |  |  |  |

AUC, area under the curve; SVC, support vector classification; LGBM, light gradient boosting machine; XGBM, extreme gradient boosting machine; LR, logistic regression.

**Supplementary Table 7. Evaluate the goodness of fit of the binary Logistic regression model using Hosmer-Lemeshow Test**

| **Step** | **Chi-square value** | **Degree of freedom** | ***P* value** |
| --- | --- | --- | --- |
| 1 | 0.000 | 0 | - |
| 2 | 0.120 | 2 | 0.942 |
| 3 | 3.809 | 5 | 0.577 |
| 4 | 6.275 | 7 | 0.508 |
| 5 | 7.983 | 7 | 0.334 |
| 6 | 12.635 | 8 | 0.125 |

**Supplementary Table 8. Evaluate the effectiveness of prediction model using the C-index**

| **Prediction model** | **C-index** | **Dxy** | **Standard deviation** | **Statistic (Z)** | ***P* value** | **N** |
| --- | --- | --- | --- | --- | --- | --- |
| Predict(mylog) | 0.891 | 0.783 | 0.050 | 15.55 | <0.001 | 230 |

**Supplementary Table 9. Evaluation of the efficacy of the validation model with different sample sizes**

| **Validation sample sizes (n)** | **Validation sample radio (%)** | **AUC** | **95%CI lower** | **95%CI upper** | **Difference value of 95%CI** |
| --- | --- | --- | --- | --- | --- |
| 69 | 30 | 0.886 | 0.741 | 0.981 | 0.240 |
| 92 | 40 | 0.828 | 0.745 | 0.902 | 0.157 |
| 115 | 50 | 0.824 | 0.715 | 0.908 | 0.193 |
| 138 | 60 | 0.782 | 0.654 | 0.891 | 0.237 |
| 161 | 70 | 0.848 | 0.684 | 0.965 | 0.281 |
| 184 | 80 | 0.878 | 0.741 | 0.973 | 0.232 |

AUC, area under the curve; CI, confidence interval.
